# Role of tertiary lymphoid structures in the tumour microenvironment and immunotherapy response of renal cell carcinoma

**DOI:** 10.1002/ctm2.70455

**Published:** 2025-10-06

**Authors:** Lin Yang, Wentai Shangguan, Weijia Li, Wenxue Huang, Zhuohang Li, Boyuan Sun, Cunzhen Ma, Xunguo Yang, Bisheng Cheng, Peng Wu

**Affiliations:** ^1^ Nanfang Hospital Southern Medical University Guangzhou China; ^2^ Department of Urology Second Affiliated Hospital of Naval Medical University Shanghai China

**Keywords:** immune checkpoint inhibitors, individualised therapy, renal cell carcinoma, tertiary lymphoid structures, tumour microenvironment

## Abstract

In the tumour microenvironment (TME) of renal cell carcinoma (RCC), tertiary lymphoid structures (TLS) play a crucial role in anti‐tumour immune responses. Resembling secondary lymphoid organs, TLS comprises B cells, T cell zones, high endothelial venules, and antigen‐presenting cells, facilitating local immune activation. While TLS has shown correlations with improved immune checkpoint inhibitors (ICIs) outcomes in other cancers, its role in RCC is still under investigation. Emerging evidence indicates that mature TLS enhances anti‐tumour activity by activating T and B cells, whereas immature TLS may contribute to immune suppression. The RCC TME is highly immunosuppressive, marked by regulatory T cells, myeloid‐derived suppressor cells, and elevated pro‐angiogenic and immunosuppressive cytokines. In this context, TLS, particularly mature TLS, can counteract immunosuppression, boost local immune responses, and improve ICIs efficacy. However, TLS in RCC is heterogeneous, with their formation and function affected by factors like CXCL13 expression. The presence, maturity, and functionality of TLS may serve as valuable predictors of ICIs response and patient prognosis. Further research is required to understand TLS regulation and leverage their potential to enhance personalised immunotherapy for RCC.

## Graphical abstract

Tertiary lymphoid structures in renal cell carcinoma exert immunoregulatory effects in the immune microenvironment by influencing local anti‐tumour immune responses, thereby affecting immunotherapy efficacy and prognosis.

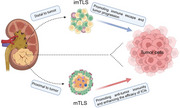

## 1 INTRODUCTION

Tertiary lymphoid structures (TLS) are lymphoid‐like formations arising in the tissue microenvironment under chronic inflammation, infection, or tumourigenesis. Structurally resembling lymph nodes and spleen, TLS formation is triggered not by normal development but by persistent inflammatory stimuli, and includes B cell and T cell zones, high endothelial venules (HEVs), and antigen‐presenting cells. TLS facilitate local immune responses via antigen presentation and immune cell activation, positioning them as key indicators of local immune activity and anti‐tumour immunity.^1^, ^2^ Studies show TLS formation is closely linked to chronic inflammation within the tumour microenvironment (TME), significantly influencing tumour immune responses. Recently, TLS has gained attention for its potential clinical relevance in solid tumours, particularly in renal cell carcinoma (RCC).^3^, ^4^

RCC, the most common kidney malignancy in adults, remains resistant to radiotherapy and chemotherapy.^5^ With advances in tumour immunology, RCC's TME has become a focus, as it reflects both anti‐tumour immune activity and notable immunosuppressive features. Immunosuppression in RCC is driven by regulatory T cells (Tregs), myeloid‐derived suppressor cells (MDSCs), and tumour‐associated macrophages, which secrete immunosuppressive cytokines such as TGF‐β and IL‐10.^6^, ^7^ Additionally, RCC's metabolic demands and hypoxic conditions activate hypoxia‐inducible factors (HIF‐1α and HIF‐2α), promoting angiogenesis and further suppressing effector T cell activity.^8^, ^9^, ^10^ RCC's high PD‐L1 expression facilitates immune evasion, making it a target for immune checkpoint inhibitors (ICIs), which have shown significant efficacy in advanced cases.^11^, ^12^, ^13^

Within the TME, TLS formation is characterised by B cells, T cells, and antigen‐presenting cells, which collectively enhance anti‐tumour immune responses. B cells in TLS undergo affinity maturation, producing high‐affinity antibodies to amplify cytotoxicity against tumour cells.^2^, ^14^ Evidence correlates TLS presence with better ICIs responses; TLS‐enriched TME displays higher immune activation and improved outcomes.^15^, ^16^ For example, in non‐small cell lung cancer (NSCLC) and melanoma, TLS correlate with enhanced ICIs response and extended survival.^17^, ^18^ Nonetheless, TLS's role in tumour immunity is context‐dependent; immature TLS (imTLS) may foster an immunosuppressive microenvironment, underscoring the need for further research to understand these dual roles.^19^, ^20^, ^21^ In RCC, TLS‐related studies are limited, though existing evidence suggests TLS may support specific anti‐tumour responses within its unique TME. Further investigation is needed to elucidate TLS mechanisms and their impact on immunotherapy in RCC.^22^, ^23^, ^24^

This review will explore TLS formation in RCC's immune microenvironment, their regulatory effects on anti‐tumour responses, their association with ICI efficacy, and potential therapeutic applications, while discussing future research directions.

## 2 FORMATION AND FUNCTION OF THE TLS

### 2.1 TLS formation mechanism

TLS formation, termed ectopic lymphogenesis, resembles the development of secondary lymphoid organs but occurs postnatally in non‐lymphoid tissues. It can be triggered by chronic or acute inflammation, depending on the tissue context, and is shaped by specific signals within the TME. Pro‐inflammatory cytokines and chemokines, notably CXCL13, CCL19, and CCL21, drive this process by recruiting lymphocytes to the tumour, where they interact with fibroblasts, endothelial cells, and stromal cells, forming organised lymphoid‐like structures.^2^, ^25^ A specialised subset of CCL19‐expressing fibroblastic reticular cells (FRCs) has been shown to orchestrate the formation of interconnected T cell zones in TLS and tumour‐associated niches. These FRCs originate from mural or adventitial precursors and support local T cell activation, differentiation, and trafficking by establishing functional niches enriched in adhesion molecules and chemokines.^26^

HEVs, a hallmark of TLS, sustain lymphocyte recruitment by expressing addressins and chemokines,^27^, ^28^ Recent evidence suggests that the maturity and activation status of HEVs directly impacts TLS functionality and antitumour immune efficacy, with well‐structured HEVs supporting compartmentalised lymphocyte infiltration and germinal centre development.^29^, ^30^ Dendritic cells (DCs), which often localise near HEVs and FRC networks, enhance antigen presentation and coordinate the spatial organisation of immune cell interactions within TLS.^1^

**FIGURE 1:**
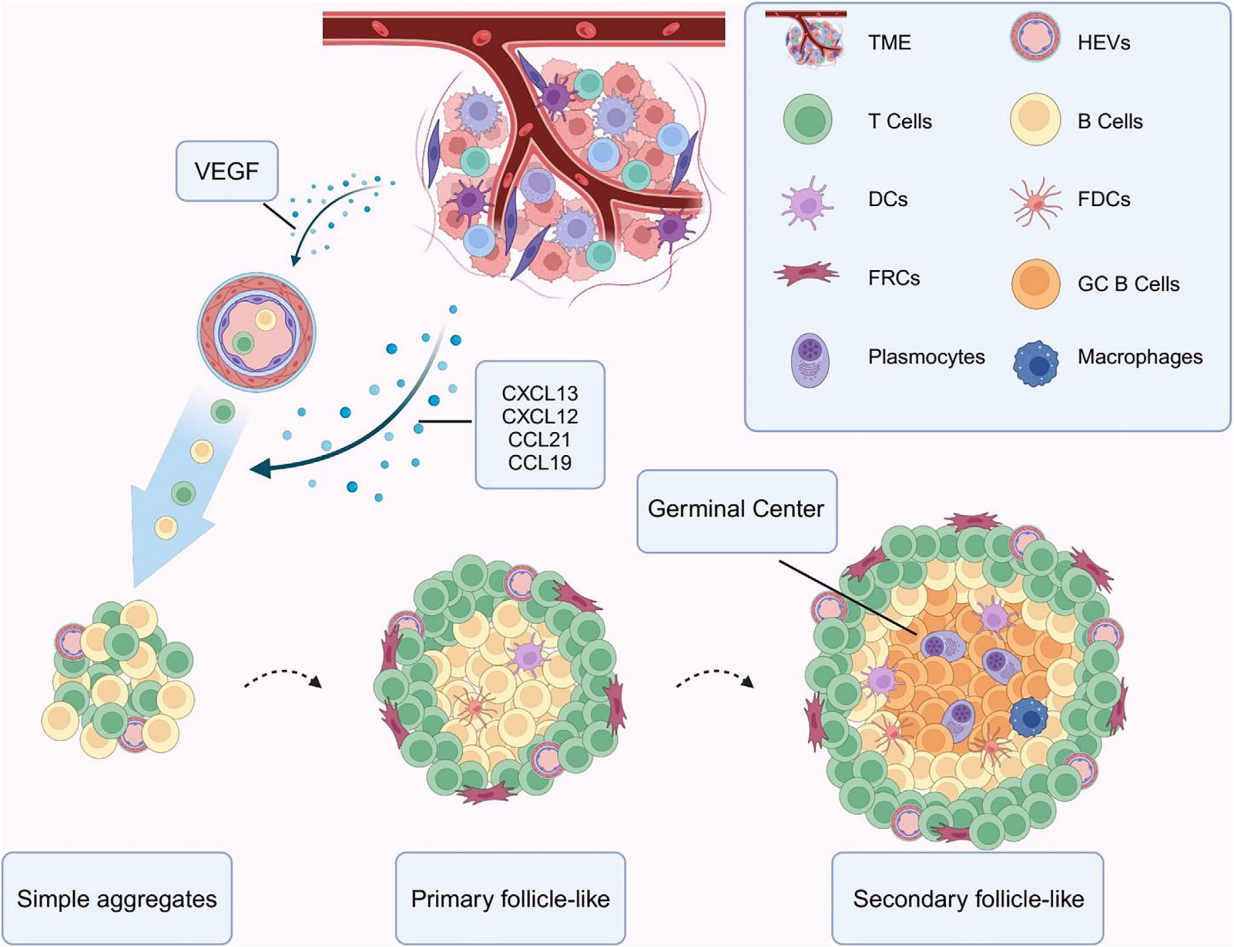
The structure and formation mechanism of TLS. In tumour tissues, TLS formation is regulated by a series of pro‐inflammatory cytokines and chemokines, especially key chemokines such as CXCL13, CXCL12, CCL19, and CCL21, which promote the migration of lymphocytes to the tumour site. VEGF promotes the generation of HEVs, which is an important marker of TLS formation and provides an important pathway for the continuous recruitment of lymphocytes. The maturation process of TLS is from early aggregation of immune cells to a highly organised structure with a germinal centre, which mainly consists of simple aggregates, primary follicle‐like TLS, and secondary follicle‐like TLS. TME, tumour microenvironment; HEVs, high endothelial veinlets; DCs, dendritic cells; FDCs, follicular dendritic cells; FRCs, fibroblastic reticular cells; GC, germinal centre; VEGF, vascular endothelial growth factor; TLS, tertiary lymphatic structures.

TLS development and maturation are also regulated by lymphotoxin αβ and tumour necrosis factor pathways, which support stromal and endothelial cell differentiation and TLS organisation.^31^, ^32^ Mature TLS progress from initial immune cell accumulation to complex structures with germinal centres where B cells and T cells become activated, enhancing both local and systemic anti‐tumour responses through ongoing antigen presentation and immune stimulation ^4^, ^16^ (Figure 1).

### 2.2 Immune function of TLS

#### 2.2.1 Immune cell composition in TLS

TLS are primarily composed of B cells and T cells. B cells cluster in germinal centre‐like areas, undergoing affinity maturation and antibody secretion, while T cells in adjacent zones engage in antigen‐specific immune responses. DCs within TLS capture and present antigens to activate T cells, with T follicular helper (Tfh) cells playing a key role in B cell maturation and antibody production. This diversity of immune cells enables TLS to act as robust immune regulatory hubs in the TME.^3^, ^33^, ^34^

#### 2.2.2 The role of TLS in antigen presentation

Positioned near tumours, TLS enhance the efficiency of antigen presentation.^16^, ^17^ TLS formation begins with the release of pro‐inflammatory chemokines, notably CXCL13, CCL19, and CCL21, which recruit B cells, naïve and central memory T cells, and DCs to the tumour site. These immune cells interact with FRCs and endothelial cells, organising into distinct lymphoid‐like aggregates. HEVs form to sustain continuous lymphocyte influx, while follicular dendritic cells (FDCs) and Tfh cells further support B cell activation, affinity maturation, and germinal centre development.^26^ TLS are generally classified by their maturity. ImTLS often present as loose aggregates lacking defined structure and germinal centres, with limited antigen presentation and weak immunologic function. In contrast, mature TLS (mTLS) are characterised by clear B cell follicles, T cell zones, germinal centre–like structures, and efficient antigen‐presenting cell networks, enabling robust activation of cytotoxic T cells and B cells.^19^, ^35^

### 2.3 Presence and heterogeneity of TLS in tumours

TLS formation and function vary significantly across tumour types, influenced by pro‐inflammatory signals, immune cell recruitment, and responsiveness to immunotherapy. In immunogenic tumours, such as NSCLC and melanoma, TLS formation is frequent and linked to improved prognosis and ICI efficacy. Here, TLS serve as platforms for antigen presentation and immune activation, enhancing T and B cell responses.^36^, ^37^, ^38^ Triple‐negative breast cancer (TNBC) similarly demonstrates improved outcomes with TLS presence, where these structures support immune cell infiltration and memory formation.^39^, ^40^

Conversely, in low‐immunogenic or ‘cold’ tumours, like pancreatic cancer and hepatocellular carcinoma, TLS are rare and often functionally impaired by a highly immunosuppressive environment with sparse immune infiltration and high levels of inhibitory cytokines (e.g., TGF‐β, IL‐10).^41^, ^42^ However, in some cold tumours, mTLS formation can still enhance anti‐tumour immunity and correlate with better clinical outcomes.^43^

Notably, in addition to tumour type and immune contexture, the spatial localisation of TLS has also emerged as a key determinant of their prognostic value. Recent studies in ccRCC demonstrate that tumour‐proximal TLS, particularly those with mature germinal centre–like structures, are significantly associated with improved progression‐free and overall survival. In contrast, tumour‐distal TLS are more likely to be immature and are often accompanied by higher levels of regulatory T cells and PD‐L1⁺ tumour‐associated macrophages, correlating with an immunosuppressive microenvironment and poorer clinical outcomes.^23^, ^44^ These findings emphasise the importance of TLS spatial distribution in predicting immunotherapy efficacy and patient prognosis. (Table 1)

**TABLE 1:** The evidence on the prognostic significance and clinical value of TLS in RCC.

Study (author/year)	Methods of TLS detection and evaluation	TLS markers	The potential function of TLS	Prognostic value of TLS	Refs
Saut 2020	H&E, IHC, and mIHC staining	B cells (CD19/20), T cells (CD3), FDCs (CD21) and HEVs (MECA79)	Supports anti‐tumour immune responses by B cells and other immune effector cells	B cells and TLS promote immunotherapy response	^4^
Masuda 2022	IHC staining	B cells (CD20), T cells (CD3), mature B cells (Bcl6/CD10) and Germinal centre plasma cells (CD21)	Compared to bladder cancer, TLS in RCC had a higher proportion of PD‐1‐positive cells and most of the TLS were in an immature state.	The presence of TLS in RCC was associated with a poorer prognosis	^21^
Meylan 2022	Spatial transcriptomics technology	12 immunoglobulin genes, 5 B cell markers, 2 T cell markers, 2 fibroblast markers, and 2 complement protein‐coding genes	TLS in tumours supports B cell maturation and antibody production	Presence of TLS is associated with better PFS and immunotherapy response in RCC	^22^
Xu 2022	Machine learning algorithms and characterisation of 12 chemokine genes	CCL4, CCL5, CCL8, CCL19 and CXCL13	TLS can reflect different TME immune status and RCC prognostic features	Elevated CXCL13 expression predicts aggressive progression and poor prognosis in RCC patients	^104^
Xu 2023	H&E, IHC, and mIHC staining	B cells (CD20), T cells (CD3), FDCs (CD21) and Germinal centre (CD23)	TLS localisation and maturation are homogeneous among RCC	The majority of TLS in RCC are located distal to the tumour and are associated with immature, immunosuppressive features, while a higher proportion of mTLS proximal to the tumour are associated with improved PFS and OS	^23^
Xu 2024	H&E, IHC, and mIHC staining	B cells (CD20), T cells (CD3), FDCs (CD21), Germinal centre (CD23), and mature DCs (DC‐LAMP)	The abundant mature plasma cells within mTLS have the ability to produce IgA and IgG, which correlates with the efficacy of ICIs	Presence of mTLS within RCC tumours correlates with better efficacy of ICIs	^44^
Wang 2024	Single‐cell RNA sequencing, IHC, and mIHC staining	B cells (CD20), T cells (CD3), FDCs and mature B cells (CD21) and HEVs (CD31)	The combination of age and TLS abundance had an impact on prognosis, with higher senescence scores detected in imTLS	Abundant mTLS infiltration in RCC was associated with better PFS, OS, and ICIs/TKI efficacy	^24^

TLS, tertiary lymphatic structures; mTLS, mature tertiary lymphoid structures; imTLS, immature tertiary lymphoid structure; RCC, renal cell carcinoma; IHC, immunohistochemistry; mIHC, multiplex fluorescence immunohistochemistry; DCs, dendritic cells; FDCs, follicular dendritic cells; HEVs, high endothelial veinlets; TME, tumour microenvironment; PD‐1, programmed cell death‐1; PFS, progression‐free survival; OS, overall survival; ICIs, immune checkpoint inhibitors; TKI, tyrosine kinase inhibitors.

## 3 TLS IN THE RCC IMMUNE MICROENVIRONMENT

### 3.1 Characteristic immune microenvironment of RCC

RCC exhibits a unique TME, distinguished by marked immunosuppression, metabolic reprogramming, and hypoxia, which collectively enable immune escape and complicate immunotherapy.^45^ Tregs suppress effector T cell function via cytokines like TGF‐β and IL‐10, while MDSCs produce reactive oxygen species and nitric oxide, directly inhibiting effector T and natural killer cells (NK cells).^46^ RCC cells rely on aerobic glycolysis, generating lactate, which lowers local pH and further promotes Treg and MDSC accumulation, establishing a strong immunosuppressive barrier.^9^, ^10^, ^47^ Hypoxia, exacerbated by rapid tumour growth, activates hypoxia‐inducible factors (HIF‐1α and HIF‐2α), driving vascular endothelial growth factor (VEGF) expression, which supports angiogenesis and, further enhancing immune evasion.^8^, ^48^, ^49^ The RCC TME's high expression of immune checkpoints, including PD‐L1 and CTLA‐4, compounds these immunosuppressive effects, explaining RCC's varied response to ICIs.^11^, ^50^, ^51^

The immune microenvironment of RCC is characterised not only by its immunosuppressive nature but also by pronounced spatial heterogeneity. Within this context, TLS serve as localised immune niches that may partially counteract immunosuppressive mechanisms. Spatial transcriptomics and single‐cell analyses have demonstrated that, in TLS‐positive RCC tumours, B cells undergo full maturation into IgG‐ and IgA‐producing plasma cells, accompanied by somatic hypermutation, clonal expansion, and local antibody secretion.^22^, ^23^ These antibodies can mediate direct tumour cell killing, as evidenced by IgG staining observed on apoptotic tumour cells, and their production has been positively correlated with durable responses to ICIs.

Moreover, a high density of TLS with robust germinal centre–like activity has been associated with prolonged progression‐free survival in patients receiving anti–PD‐1 therapy. In contrast, enrichment of tissue‐resident exhausted CD8⁺ T cells (e.g., ZNF683⁺SLAMF7⁺) correlates with poor clinical outcomes, underscoring the immunosuppressive forces within the RCC microenvironment.^52^ Notably, unlike most solid tumours where CD8⁺ tumour‐infiltrating lymphocyte (TIL) infiltration predicts favourable prognosis, RCC displays a unique immunological paradox in which high CD8⁺ TIL content is often associated with T cell dysfunction and worse outcomes.^53^ This complexity suggests that the prognostic and therapeutic value of TLS in RCC is context‐dependent and may be shaped by the relative abundance and functional state of co‐infiltrating immune populations. Thus, a TLS‐rich yet low‐exhaustion immune milieu may represent the optimal landscape for ICI responsiveness.

### 3.2 Presence and role of TLS in the RCC immune microenvironment

Recent studies have revealed significant heterogeneity in the formation, spatial distribution, and clinical significance of TLS in RCC across different patient subgroups and histological subtypes. In the most common subtype – clear cell RCC (ccRCC) – mature intratumoural TLS have been confirmed by multiple large‐scale, multi‐centre cohort and spatial transcriptomic studies to be associated with improved survival outcomes and stronger responses to PD‐1/PD‐L1 immune checkpoint blockade.^21^, ^23^, ^24^ These TLS are enriched with functional plasma cells expressing IgA/IgG, suggesting their role in supporting an active antitumour immune microenvironment.^22^ In contrast, immature or peritumoural TLS in ccRCC are frequently accompanied by infiltrates of regulatory T cells (Tregs) and PD‐L1–positive tumour‐associated macrophages, indicating an immunosuppressive phenotype and correlating with poorer clinical outcomes.^23^

Of particular note, the heterogeneity of TLS among different RCC subtypes is becoming increasingly evident. For example, in SMARCB1‐deficient medullary RCC – a rare and aggressive subtype – TLS also show prognostic value. A small cohort study reported that mature TLS were associated with favourable responses to ICI and tyrosine kinase inhibitor combination therapy. Conversely, papillary RCC and chromophobe RCC exhibit lower frequencies of TLS, and their immune microenvironments may be less supportive of TLS‐mediated antitumour immunity, although further research in this area is still limited.^54^

### 3.3 Immunomodulation of TLS in the TME of RCC

#### 3.3.1 Promoting anti‐tumour immune response

**FIGURE 2:**
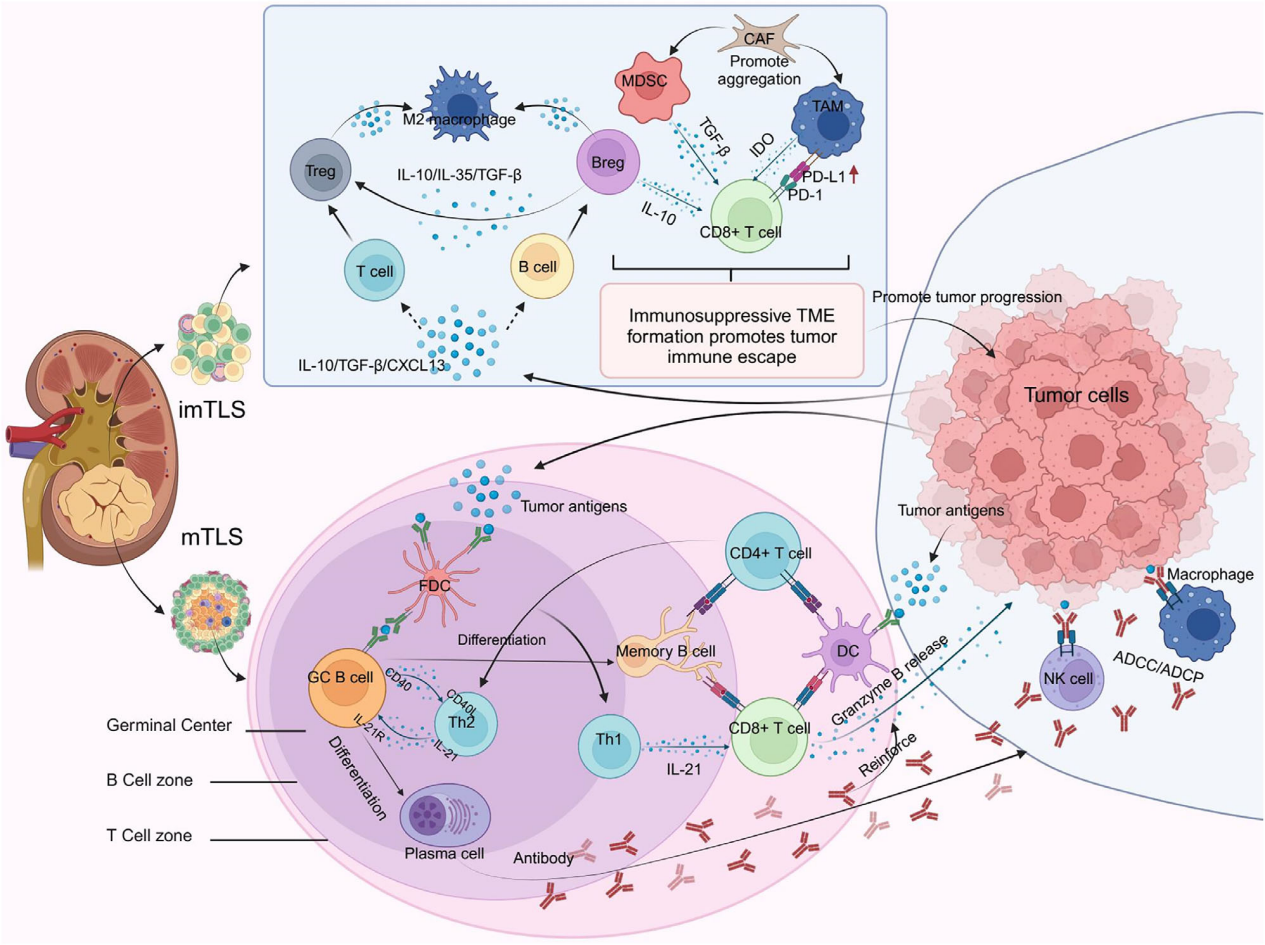
The Dual Role of TLS in RCC. ImTLS structures often lack effective immune cell organisation and function to activate an adequate anti‐tumour immune response. Instead, they are able to recruit immunosuppressive cells such as Tregs, Bregs, and MDSCs, which are capable of not only directly suppressing effector T‐cell function by expressing immune checkpoint molecules in the TME, but also secreting cytokines such as IL‐10, IL‐35, and TGF‐β to inhibit anti‐tumour immune responses. In mTLS, DCs activate specific anti‐tumour immune responses by capturing tumour antigens and presenting them to CD4+ and CD8+ T cells. CD8+ T cells are the main effector cells, and their activation enables them to directly recognise and kill tumour cells, creating a potent immune defence. This tumour‐killing effect depends on the recognition of MHC and TCR. B cells, supported by FDCs, generate high‐affinity antibodies through somatic hypermutation and affinity maturation processes, which not only neutralise tumour antigens directly, but also activate NK cells and macrophages, and destroy tumour cells through the ADCC or ADCP pathways. In addition, the interaction between T cells and B cells further enhances the immune response. ImTLS, immature tertiary lymphoid structures; Tregs, regulatory T cells; Bregs, regulatory B cells; MDSCs, myeloid‐derived suppressor cells; TAM, tumour‐associated macrophages; CAF, cancer‐associated fibroblast; IDO, indoleamine 2,3‐dioxygenase; PD‐1, programmed death receptor 1; PD‐L1, programmed death ligand 1; mTLS, mature tertiary lymphoid structures; MHC, major histocompatibility complex; TCR, T cell receptor; FDCs, follicular dendritic cell; NK cells, natural killer cell; ADCC, antibody‐dependent cell‐mediated cytotoxicity; ADCP, antibody‐dependent cellular phagocytosis.

TLS serve as local immune hubs, B cells within TLS, supported by FDCs, produce high‐affinity antibodies that facilitate tumour cell destruction through antibody‐dependent cytotoxicity and phagocytosis ^4^, ^22^, ^55^ (Figure 2). Interactions between T and B cells in TLS further enhance immune response and contribute to long‐term immune memory, improving local tumour surveillance and aiding in recurrence prevention.^56^, ^57^

#### 3.3.2 Modulation of immunosuppressive factors

TLS also attract immunosuppressive cell subsets, such as Tregs, Bregs, and MDSCs, which limit anti‐tumour immunity through multiple mechanisms, including the secretion of suppressive cytokines (e.g., IL‐10, TGF‐β, IL‐35) and the expression of checkpoint molecules such as CTLA‐4 and PD‐1.^20^, ^58^ Within TLS, Tregs are primarily located in the T cell zones, where they suppress local CD4⁺ and CD8⁺ T cell proliferation and function via CTLA‐4‐mediated dendritic cell inhibition and CD39‐driven adenosine production.^59^, ^60^ High Treg density in TLS has been associated with reduced TLS‐mediated immunosurveillance and poor prognosis, as shown in NSCLC cohorts. In addition, follicular regulatory T cells, a CXCR5⁺FOXP3⁺ subset localised within B cell follicles, can inhibit Tfh cells and dampen B cell responses, thereby impairing TLS maturation. Breg cells, producing IL‐10, may further contribute to the immunosuppressive microenvironment within TLS, especially in immature TLS lacking organised germinal centres.^61^, ^62^ The relative abundance and spatial distribution of these regulatory subsets within TLS critically influence their structure and function, and an imbalance favouring immunosuppression can convert TLS into permissive niches for tumour progression.

## 4 TLS AND IMMUNOTHERAPEUTIC RESPONSE

### 4.1 Impact of TLS on the effectiveness of immunotherapy

#### 4.1.1 Effectiveness of ICIs in TLS‐rich tumours

**FIGURE 3:**
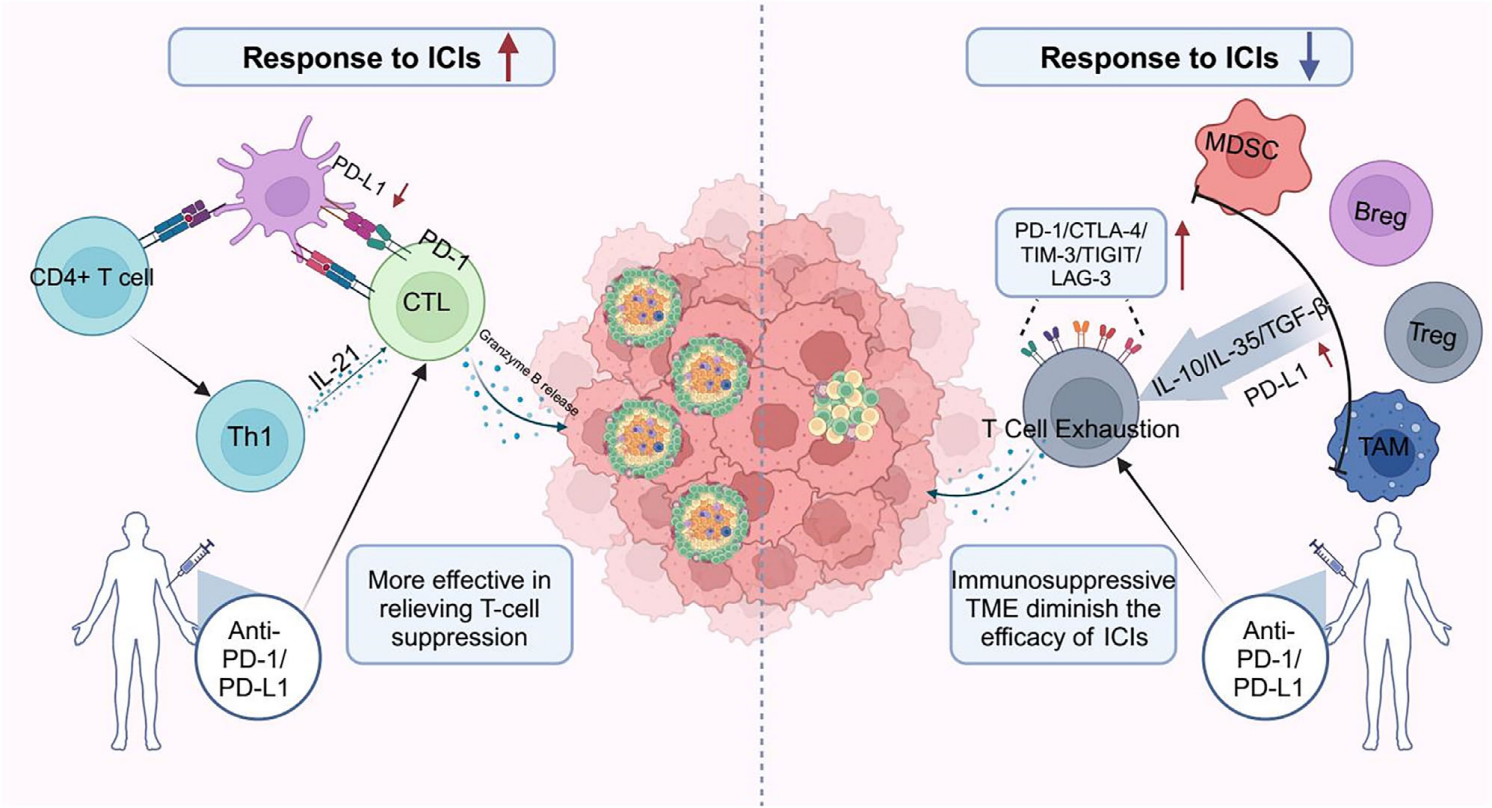
Relationship between TLS and the efficacy of ICIs. Patients with TLS‐rich tumours typically have a stronger therapeutic response to PD‐1 or PD‐L1 inhibitors. DCs in the TLS present tumour antigens to CD4+ and CD8+ T cells within the TLS by capturing and processing them. This process not only effectively activates effector T cells, but also enhances the cytotoxic response of T cells by promoting the secretion of Th1‐type cytokines. This local immune activation effect allows PD‐1/PD‐L1 inhibitors to more efficiently deregulate T cells and prompt effector T cells to continuously attack tumour cells. In tumour patients lacking TLS, a large number of immunosuppressive cells accumulate in the TME, resulting in an immunosuppressive microenvironment, which inhibits the function of effector T cells and leads to poor efficacy of PD‐1/PD‐L1 inhibitors. ICIs, immune checkpoint inhibitors; Th1, helper T cell 1; CTL, cytotoxic T cell.

The presence of TLS within the TME significantly enhances anti‐tumour immune responses, particularly in the context of ICIs and CAR‐T therapies. In mTLS‐rich tumours, PD‐L1 expression is typically lower or spatially restricted within TLS areas, reducing immune inhibition and improving ICI responsiveness, as evidenced by prolonged progression‐free (PFS) and overall survival (OS) ^63^ (Figure 3). Within mature TLS, DCs capture tumour antigens and activate both CD4⁺ helper T cells and CD8⁺ cytotoxic T cells, which differentiate into effector cells capable of producing IFN‐γ and mediating direct tumour killing.^16^ Concurrently, naïve B cells undergo affinity maturation and class switching upon interaction with Tfh cells and FDCs, giving rise to plasma cells that secrete high‐affinity antibodies. These antibodies mediate antibody‐dependent cellular cytotoxicity and complement‐dependent cytotoxicity. This spatial proximity enables rapid and localised immune priming while circumventing lymph node trafficking, thereby intensifying both cellular and humoral anti‐tumour responses.^4^, ^63^

In addition to their presence and maturity, the spatial localisation of TLS has emerged as a key determinant of their immunologic impact. Intra‐tumoural TLS, especially those with germinal centre–like features, are linked to enhanced effector lymphocyte activity and favourable responses to anti–PD‐1 therapy. In contrast, peri‐tumoural TLS, often immature and surrounded by regulatory T cells and immunosuppressive macrophages, correlate with limited ICI benefit and poorer clinical outcomes.^23^, ^44^ These findings underscore the importance of considering both maturation status and spatial distribution when evaluating TLS as biomarkers or therapeutic modulators in RCC.

#### 4.1.2 Potential synergies between TLS and CAR‐T cell therapy

CAR‐T cell therapy involves genetically modifying patients' T cells to enhance their ability to target specific tumour antigens with potent cytotoxicity.^64^ CAR‐T cell efficacy in solid tumours is often limited by the immunosuppressive TME, but TLS can enhance CAR‐T function by supporting local immune activation. TLS provide continuous antigen stimulation and immune support, extending CAR‐T cell persistence and activity.^3^, ^65^, ^66^ Additionally, TLS can help remodel the immunosuppressive tumour microenvironment by reducing the presence or activity of Tregs, MDSCs, and immunosuppressive macrophages, thereby enhancing the infiltration, expansion, and cytotoxicity of CAR‐T cells.^67^ Recent studies suggest that TLS‐inducing factors, such as LIGHT, can promote CCL19/CCL21 expression, normalise tumour vasculature, and facilitate the formation of lymphoid‐like structures, collectively improving CAR‐T cell trafficking and function.^67^, ^68^, ^69^ These findings indicate that targeting TLS formation or incorporating TLS‐promoting cytokines into CAR‐T constructs, such as LIGHT‐CAR‐T cells, may overcome the immunosuppressive barriers in solid tumours and enhance therapeutic outcomes.

### 4.2 TLS as a prognostic marker of immunotherapy efficacy

#### 4.2.1 Prognostic predictive role of TLS in immunotherapy

TLS density, spatial localisation, and maturation status are closely associated with prognosis and immunotherapy efficacy in RCC. MTLS are structurally characterised by distinct B cell follicles, T cell zones, FDCs, and HEVs, which together support efficient local antigen presentation and immune activation. These structures harbour B cells that undergo somatic hypermutation and clonal expansion, eventually differentiating into IgG‐ and IgA‐producing plasma cells. Such plasma cells disseminate into tumour beds and contribute to anti‐tumour responses via antibody‐mediated mechanisms, as demonstrated by spatial transcriptomic and histological studies in ccRCC patients receiving ICIs.^42^, ^70^

In contrast, imTLS, which lack organised germinal centres and often reside in peritumoural regions, are frequently enriched with Tregs, tumour‐associated macrophages, and exhausted CD8⁺ T cells. These immunosuppressive components, along with the limited functional architecture of imTLS, contribute to suboptimal antigen presentation and dampened effector responses, correlating with reduced sensitivity to ICIs and inferior survival outcomes.^19^, ^21^

#### 4.2.2 Integrating TLS with other immune markers

Combining TLS analysis with other immune markers, such as PD‐L1 expression, tumour‐infiltrating lymphocytes (TILs), and exhaustion markers (e.g., TIM‐3, LAG‐3), improves predictive accuracy for immunotherapy response. PD‐L1 combined with mTLS density enhances the predictive value for ICI response in RCC.^15^ The abundance of TILs in TLS‐rich tumours is positively correlated with immunotherapy efficacy, as TLS and TILs together indicate a robust immune environment.^71^ Additionally, mTLS can mitigate T cell exhaustion, suggesting that combining mTLS analysis with exhaustion markers could further refine immunotherapy predictions and guide personalised treatments.^72^

## 5 TLS REGULATORY MECHANISMS IN RCC

### 5.1 Molecular mechanisms promoting TLS formation

#### 5.1.1 Chemokine CXCL13 and TLS formation

TLS formation involves interactions among various chemokines and immune cells, with CXCL13 playing a critical regulatory role. CXCL13 is primarily secreted by FDCs, fibroblasts, activated B cells, and macrophages, and binds to CXCR5 on lymphocytes, directing the migration and organisation of CXCR5⁺ B cells and T cells into TLS.^73^ In many tumours, elevated CXCL13 expression is linked to enhanced TLS formation and improved immune activation.^74^, ^75^ However, in RCC, CXCL13 may exert dual immunological effects. In addition to its role in supporting TLS assembly and B cell maturation, recent studies have identified a population of CXCL13⁺CD8⁺ T cells within the intratumoural compartment of ccRCC that exhibit an exhausted phenotype, characterised by upregulation of PD‐1, TIGIT, TIM‐3, and CTLA‐4, along with reduced IFN‐γ and TNF‐α expression.^76^ These cells are associated with an immunoevasive tumour microenvironment enriched in Tregs, Th2 cells, and tumour‐associated macrophages, and correlate with poorer prognosis and impaired cytotoxic CD8⁺ T cell function.^21^, ^77^

These findings highlight that the impact of CXCL13 in RCC depends not only on its cellular source but also on the immune contexture of the tumour microenvironment. CXCL13 derived from stromal or lymphoid organiser cells may facilitate protective TLS assembly, while CXCL13‐producing exhausted CD8⁺ T cells may reflect or reinforce an immunosuppressive milieu. Therefore, therapeutic strategies aiming to modulate CXCL13 should consider co‐targeting inhibitory pathways (e.g., PD‐1, TIGIT) or selectively enhancing its expression in pro‐immunogenic cell populations to promote TLS maturation without exacerbating immune exhaustion.

#### 5.1.2 CXCR5‐positive B cells in TLS

CXCR5‐positive B cells are essential in TLS formation and maintenance, responding to CXCL13 by forming B cell follicles within TLS. In TLS, these B cells undergo somatic hypermutation and affinity maturation, producing tumour‐specific antibodies and promoting effector T cell activation through antigen presentation.^4^, ^20^, ^78^ In RCC, TLS enriched with CXCR5‐positive B cells correlate with strong anti‐tumour responses and heightened immunotherapy efficacy.^20^, ^79^ Collaborative interactions with Tfh cells, DCs, and FDCs support local immune surveillance and sustain immune responses.^80^, ^81^

### 5.2 Factors affecting TLS formation and function

#### 5.2.1 Tumour cell secretions and TLS

TLS development and functionality are influenced by tumour‐secreted factors, tumour stroma, and other components within the TME. Pro‐inflammatory cytokines, especially IFN‐γ, promote TLS formation by activating DCs and upregulating CXCL13 expression, enhancing immune cell recruitment and activity.^82^ In contrast, VEGF, a pro‐angiogenic molecule abundantly expressed in RCC, impairs TLS development by disrupting HEVs formation, thereby limiting lymphocyte infiltration and antigen presentation within TLS. Recent preclinical studies have demonstrated that VEGF inhibition, especially when combined with PD‐L1 blockade, can restore HEVs formation through activation of the LTβR signalling pathway, subsequently enhancing TLS development, cytotoxic T cell infiltration, and tumour cell apoptosis.^83^ These findings suggest that anti‐VEGF therapy not only normalises tumour vasculature but also creates a permissive immune niche for TLS maturation, especially when integrated with immune checkpoint inhibitors. Additionally, RCC's metabolic shift towards glycolysis produces lactate, which acidifies the TME, inhibits DC maturation, and impairs TLS immune functions, underscoring the potential of targeting tumour metabolism to enhance TLS efficacy.^83^, ^84^

#### 5.2.2 Tumour stroma and microenvironment regulation

The physical and chemical properties of the TME – including fibroblasts, extracellular matrix (ECM), and oxygen levels – critically regulate TLS formation and functionality. Cancer‐associated fibroblasts (CAFs) in RCC secrete chemokines such as CXCL12 to recruit immune cells and sustain TLS formation, while also releasing TGF‐β, which can suppress TLS maturation and function.^85^ Moreover, recent studies have identified a specialised subset of fibroblasts resembling FRCs, characterised by CCL19 and podoplanin (PDPN) expression, which form cellular ‘tracks’ that direct T cell migration and organisation within TLS.^26^ These FRC‐like stromal cells create supportive niches that promote TLS maturation and effective T cell activation. In addition, the ECM provides essential structural support for TLS development. Proper matrix composition facilitates immune cell retention and compartmentalisation, whereas excessive ECM deposition or fibrosis can impair immune cell trafficking and disrupt TLS architecture.^86^, ^87^

## 6 CLINICAL APPLICATIONS OF TLS AND RCC IMMUNOTHERAPY

### 6.1 TLS as a target in immunotherapy strategies

#### 6.1.1 Activating TLS to enhance immunotherapy

TLS play a crucial role in anti‐tumour immunity by supporting antigen presentation, T cell activation, and immune memory. Enhancing TLS formation may improve immunotherapy outcomes, as RCC patients with high‐density mTLS often show better responses and prolonged OS with PD‐1 or PD‐L1 inhibitors.^22^, ^24^, ^44^ This suggests that TLS could serve as both a biomarker for immunotherapy response and a therapeutic target to increase treatment efficacy by reinforcing immune activation within the TME.

#### 6.1.2 Combination therapy: TLS modulators and ICIs

Combining TLS modulators with ICIs, such as PD‐1/PD‐L1 and CTLA‐4 inhibitors, could enhance therapeutic responses in RCC. Preclinical studies show that agents like CXCL13 or IL‐36, when combined with ICIs, amplify anti‐tumour effects by promoting T cell infiltration and activation.^88^, ^89^ Clinical trials combining VEGF inhibitors, such as bevacizumab, with PD‐1 inhibitors have shown promising results in RCC, improving PFS and OS.^90^, ^91^ Despite these advances, TLS heterogeneity across patients remains a challenge, underscoring the need for prognostic biomarkers to guide personalised treatment strategies.

**FIGURE 4:**
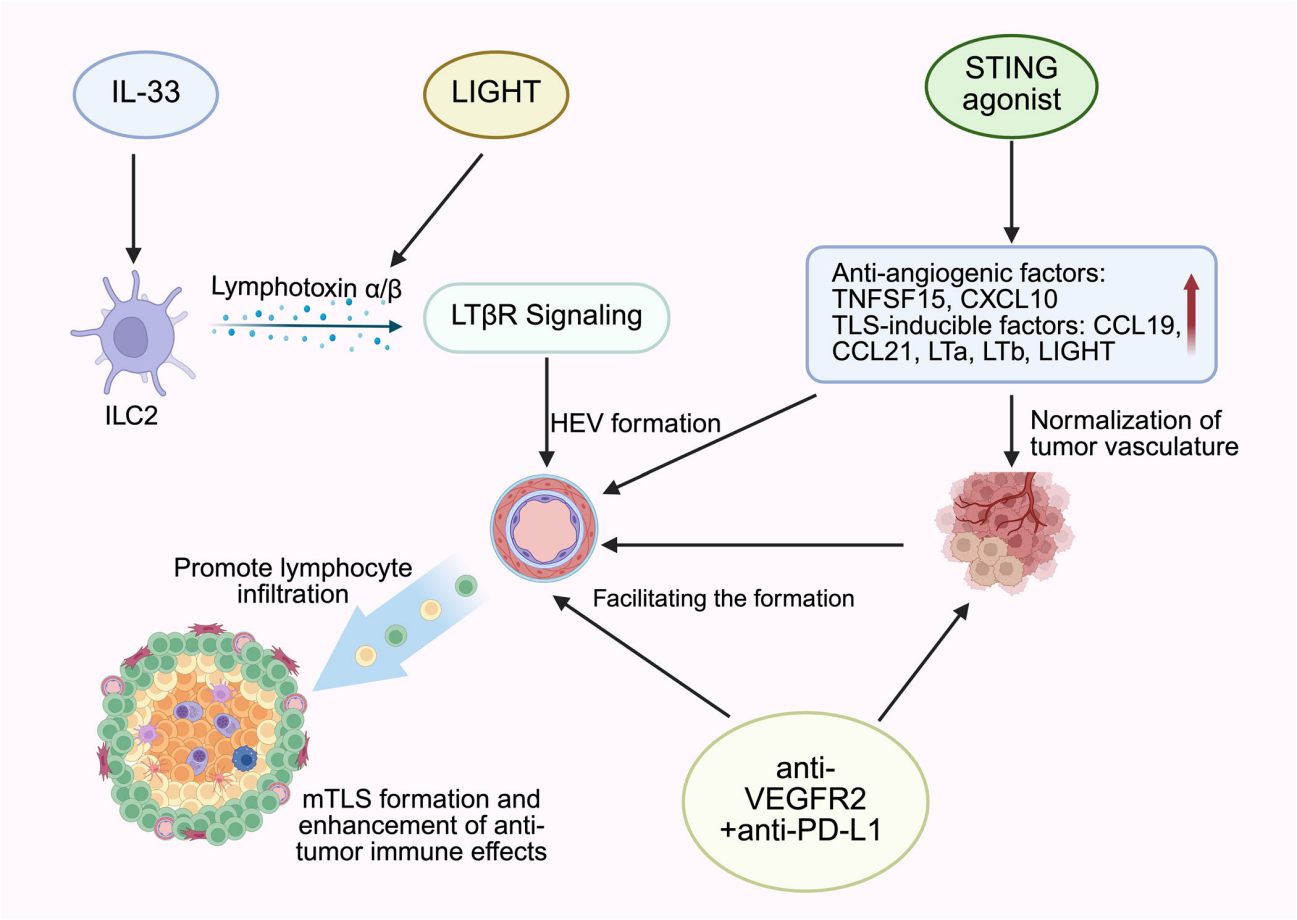
Mechanistic synergy between TLS‐inducing factors and immune checkpoint therapy. Strategies that promote the formation of HEVs in tumours can drive the development of mTLS, thereby improving immune cell infiltration and antitumour responses. Multiple pathways – including IL‐33–ILC2–LTβR, LIGHT cytokine delivery, STING activation, and antiangiogenic therapy combined with immune checkpoint inhibitors – have been shown to induce HEV formation. These HEVs recruit naïve lymphocytes into the tumour microenvironment and organise local immune structures, facilitating antigen presentation and sustained cytotoxic T cell activity. Therapeutic induction of HEV and mTLS thus reprograms the tumour microenvironment into an immune‐reactive niche, enhancing the efficacy of PD‐1/PD‐L1 blockade and other immunotherapies. HEVs, high endothelial venules; ILC2, group 2 innate lymphoid cells; STING, stimulator of interferon genes.

The latest findings suggest that IL‐33 as a novel TLS‐inducing cytokine with translational relevance. Released by inflamed tissues, IL‐33 activates group 2 innate lymphoid cells (ILC2s), which produce lymphotoxin and engage lymphotoxin β receptor (LTβR)⁺ myeloid cells to initiate lymphoneogenesis. While most mechanistic insights derive from models of pancreatic cancer and colitis, IL‐33⁺ stromal cells and lymphoneogenic ILC2s have also been detected in human tumours and are associated with increased TLS formation and improved prognosis. Recombinant IL‐33 has been shown to expand intratumoural TLSs and ILC2 populations, enhancing anti‐tumour immunity in preclinical studies.^92^ These findings suggest that the IL‐33–ILC2–LTβR axis may represent a promising target to promote TLS development and improve ICI responsiveness in RCC, warranting further investigation in RCC‐specific settings (Figure 4).

### 6.2 Potential therapeutic strategies to modulate TLS

#### 6.2.1 Promoting TLS formation

Targeting TLS formation via drugs or gene therapy to boost anti‐tumour immunity is a promising approach. Increasing CXCL13 or IL‐36 levels has demonstrated success in enhancing TLS formation in preclinical models.^93^, ^94^ Targeting the lymphotoxin β receptor signalling pathway also promotes TLS development, which may further enhance immunotherapy efficacy.^95^ However, precise regulation of these factors is necessary to avoid excessive immune activation and potential autoimmune effects, requiring careful dosing and timing for clinical application.^96^

#### 6.2.2 Modulating TLS microenvironment

TLS function can be further improved by adjusting the TME, such as by reducing lactate accumulation, which inhibits TLS immune responses. Lactate inhibitors, such as dichloroacetate, have shown potential to enhance immune responses in preclinical models.^97^ Modulating the ECM and tumour stroma, particularly through CAF‐targeting agents, may also facilitate immune cell infiltration and support TLS formation.^85^, ^86^, ^98^ Nonetheless, safely targeting these pathways in humans requires further research.

### 6.3 Clinical studies and prospects of TLS in the treatment of RCC

#### 6.3.1 TLS‐related clinical trials

Growing clinical evidence supports the predictive value of TLS, particularly mature intertumoural TLS, in enhancing immunotherapy efficacy across multiple solid tumours. In RCC, a phase II clinical trial of nivolumab (NCT03117309) involving 102 patients revealed that responders exhibited enrichment of TLS‐associated gene signatures, and a low abundance of exhausted tissue‐resident CD8⁺ T cells correlated with better outcomes.^52^ Spatial transcriptomic analyses further demonstrated that intertumoural TLS sustain B cell maturation into IgG‐ and IgA‐producing plasma cells, which contribute to tumour cell clearance and correlate with prolonged progression‐free survival in ICI‐treated patients.^22^ Additionally, in a cohort of 655 ccRCC patients, intertumoural secondary follicle‐like TLS (SFL‐TLS) were associated with favourable responses to PD‐1/PD‐L1 blockade, while peritumoural early TLS coincided with immunosuppressive microenvironments and inferior outcomes.^44^ Though direct TLS‐targeted clinical trials remain scarce, these studies collectively highlight TLS maturation and spatial distribution as promising biomarkers for response prediction and personalised immunotherapy in RCC.

#### 6.3.2 TLS in personalised therapy

TLS offers significant potential for personalised immunotherapy by refining immune profiling within the TME. Beyond mere presence, factors such as TLS density, maturation status, spatial distribution, and molecular subtype are emerging as critical parameters for clinical stratification.

Mao et al. conducted a multicentre cohort study in colorectal cancer, revealing significant differences in TLS density between right‐ and left‐sided tumours, with higher abundance and prognostic value observed in right‐sided cancers. They developed TLS‐integrated nomograms that outperformed traditional AJCC staging systems in predicting survival, underscoring the clinical utility of TLS for subtype‐specific prognosis and treatment planning.^99^ In another large‐scale study using multi‐omics and digital pathology, researchers identified three transcriptionally distinct TLS patterns in colorectal cancer, each linked to unique biological behaviours and therapeutic responses. A quantitative TLSscore, derived from core TLS signature genes, was developed to capture individual TLS profiles. This score demonstrated predictive value for both immunotherapy and chemotherapy outcomes. Notably, patients with low TLSscore – indicative of immune‐activated TLS subtypes – exhibited better prognosis and greater benefit from immune checkpoint inhibitors, whereas high TLSscore patients responded more favourably to chemotherapy.^100^

While similar TLS‐based classifiers have yet to be established in RCC, emerging technologies such as spatial transcriptomics, single‐cell analysis, and digital pathology are laying the groundwork for their development. Integrating TLS profiling with additional immune markers – such as PD‐L1 expression, tumour‐infiltrating lymphocytes (TILs), and T cell exhaustion signatures – will enhance the precision of immunotherapy for RCC patients with diverse immune landscapes.

## 7 PROSPECTS AND CHALLENGES

### 7.1 The potential of TLS in RCC tumour immunotherapy

The potential of TLS in RCC immunotherapy is primarily reflected in its role as a prognostic biomarker, therapeutic target, and its synergistic effects with ICIs. By assessing TLS density and maturity, clinicians can better stratify patients and develop personalised treatment plans based on the presence of TLS. To advance the clinical application of TLS in RCC immunotherapy, future research needs to address several key issues.

Although TLS density and maturity have been shown to correlate with immune therapy responses, methods for their detection and quantification have not yet been standardised. Future studies should focus on developing more precise TLS detection techniques, such as imaging, biomarker analysis, or high‐throughput sequencing, to accurately assess TLS status in different patients. Additionally, the formation and function of TLS are influenced by various TME factors, including chemokines, cytokines, and tumour metabolism. Future research could delve deeper into the mechanisms by which TLS operates across different tumour stages and patient populations, particularly in how these factors can be modulated to enhance TLS functionality. Furthermore, the development and optimisation of TLS modulators will be a crucial step in advancing its clinical application. More importantly, to integrate TLS into the standard protocol for RCC immunotherapy, large‐scale, multicentre clinical trials will be needed to validate the feasibility of TLS as a prognostic biomarker and therapeutic target. These studies will provide robust clinical evidence to support the widespread use of TLS in personalised treatment.

### 7.2 Technical and methodological challenges in TLS research

Despite the growing recognition of the potential of TLS in RCC immunotherapy, related research faces numerous technical and methodological challenges. As a dynamic structure of the TME, the complex regulatory mechanisms and heterogeneity of TLS present significant difficulties in the study. Currently, TLS detection primarily relies on pathological techniques such as immunohistochemistry and multiplex staining methods. However, the heterogeneity of TLS results in significant variations in TLS formation, density, and maturity across different tumours, standardised definitions and quantification methods for TLS are yet to be fully established. Future studies will need to develop more sensitive and precise detection methods to standardise TLS assessment across different tumour types.^101^ Despite the growing recognition of the potential of TLS in RCC immunotherapy, related research faces numerous technical and methodological challenges. As a dynamic structure of the TME, the complex regulatory mechanisms and heterogeneity of TLS present significant difficulties in the study. Currently, TLS detection primarily relies on pathological techniques such as immunohistochemistry and multiplex staining methods. However, the heterogeneity of TLS results in significant variations in TLS formation, density, and maturity across different tumours, standardised definitions and quantification methods for TLS are yet to be fully established. Future studies will need to develop more sensitive and precise detection methods to standardise TLS assessment across different tumour types.

TLS contains a variety of immune cells, and the functionality of these cells can vary significantly across different tumour types, stages, and patients. Thus, studying the function of TLS faces complex issues related to the regulation of intercellular signalling networks. Investigating the interactions of immune cells within TLS will require more refined single‐cell sequencing techniques and spatial transcriptomics approaches.^71^ These will help decode the functional differentiation states of immune cells within TLS and how they regulate anti‐tumour immune responses. Moreover, the heterogeneity of TLS poses a major challenge for its clinical application. Variations in density, spatial distribution, and structural maturity – from imTLS to mature (mTLS) forms – contribute to diverse immunological effects. While mTLS are typically linked to active anti‐tumour immunity and favourable outcomes, the role of imTLS is more nuanced. Emerging evidence suggests that imTLS may contribute to immunosuppression or tolerance in certain contexts, though classifying them as uniformly suppressive is likely an oversimplification.^21^ In vivo, imTLS and mTLS often coexist within the 3D tumour microenvironment, where their collective impact depends on relative abundance, spatial organisation, and crosstalk with surrounding immune cells. This complexity underscores the need for high‐resolution 3D imaging and spatial omics to map TLS maturation states and assess their integrated influence on therapy response and prognosis in RCC.

Recent studies have utilised transcriptomic and single‐cell sequencing data to identify TLS‐related markers and therapeutic targets in solid tumours. For example, in breast cancer, researchers integrated bulk transcriptome and single‐cell data to identify LGALS2 as a key molecule expressed by TLS‐associated dendritic cells, with implications for TLS‐mediated immune activation and patient stratification.^102^ Furthermore, transcriptomic data screening combined with extensive experimental validation has demonstrated that IL‐33 promotes TLS formation by activating lymphoneogenic ILC2s, providing a novel and druggable pathway to induce TLS and enhance anti‐tumour immunity.^92^ These examples highlight the growing role of genomics‐driven TLS analysis in identifying candidate regulators for future immunotherapeutic interventions.

### 7.3 Interaction of TLS with other TME components and future research directions

TLS, as an essential component of the TME, is regulated not only by the internal immune cells but also by the tumour stroma, VEGF, and other microenvironmental factors. CAFs, a key component of the tumour stroma, influence the formation and development of TLS by secreting cytokines (e.g., CXCL12, TGF‐β), and ECM components search should focus on investigating how CAFs regulate the formation and function of TLS, alter immune cell infiltration patterns, and promote the functional maturation of TLS through tumour stroma remodelling.^87^ In addition, researchers could use in vitro tumour organoid models, animal models, and spatial transcriptomics analysis to explore the dynamic interactions between CAFs and TLS.

The interaction between TLS and angiogenesis is primarily regulated by VEGF. Elevated VEGF levels promote disorganised angiogenesis and concurrently suppress the formation of HEVs, thereby obstructing lymphocyte infiltration into TLS and impairing local antigen presentation and T cell activation. Notably, recent preclinical studies have shown that combined inhibition of VEGFR2 and PD‐L1 can synergistically promote HEV formation in tumour tissues, particularly through the activation of the LTβR signalling pathway.^83^, ^95^ This structural remodelling enhances cytotoxic T cell infiltration and tumour cell apoptosis, ultimately sensitising otherwise resistant tumours to immunotherapy. These findings suggest that VEGF blockade may not directly induce TLS but rather normalise tumour vasculature and create a permissive microenvironment for HEV and TLS development. Therefore, combining VEGF inhibitors with ICIs has shown promising effects in both clinical and preclinical studies and may become part of the future standard of care in RCC and other solid tumours.^91^

In addition, lactic acid accumulated in TME inhibits immune cell activity, especially the activation of DCs and effector T cells in TLS by lowering the local pH.^103^ Future studies should focus on how metabolites, and hypoxia, inhibit the anti‐tumour function of TLS by affecting the activity of immune cells inside the TLS. Meanwhile, through metabolic interventions, such as the use of lactate metabolism inhibitors or hypoxia adaptive modulators, it can be explored how to reverse the metabolic inhibitory effects on TLS.

## 8 CONCLUSION

TLS plays an important immunoregulatory role in the immune microenvironment of RCC, promoting antigen presentation, T cell activation, and maintenance of immune memory. The density and maturation of TLS are closely related to the response to immunotherapy and prognosis of RCC patients, especially the synergistic effect with ICIs, which enhances the intensity of local immune responses. As an emerging target for RCC immunotherapy, TLS exhibits significant therapeutic potential by enhancing local anti‐tumour immune responses. The presence of TLS not only serves as a prognostic marker for evaluating the response to immunotherapy but also offers the possibility of developing new immunotherapy combinatorial strategies, such as combining with VEGF inhibitors or TLS modulators, to optimise the treatment regimen for patients. Future studies should focus on exploring the regulatory mechanisms of TLS, especially its interactions with other components of the TME, and identifying the key regulators that affect TLS formation and function. In addition, the development of TLS modulators and the application of TLS in personalised immunotherapy will be an important direction to improve the immunotherapy effect of RCC.

## AUTHOR CONTRIBUTIONS

LY, WTSG, WJL: writing – original draft. WXH, ZHL, BYS: drawing figures. CZM, XGY: literature search, tabulation. BSC, PW: writing – review &editing.

## CONFLICT OF INTEREST STATEMENT

We declare that none of the authors have any competing interests.

## ETHICS APPROVAL AND CONSENT TO PARTICIPATE

Not applicable as this is a review article. No human or animal subjects were involved in this study by the authors.

## CONSENT FOR PUBLICATION

All authors have approved the publication of this paper.
